# Clinical effectiveness of a multitarget urine DNA test for urothelial carcinoma detection: a double-blinded, multicenter, prospective trial

**DOI:** 10.1186/s12943-024-01974-4

**Published:** 2024-03-19

**Authors:** Junlong Wu, Yuda Lin, Kaiwei Yang, Xiao Liu, Huina Wang, Tingting Yu, Ran Tao, Jing Guo, Libin Chen, Huanqing Cheng, Feng Lou, Shanbo Cao, Wei Yu, Hailong Hu, Dingwei Ye

**Affiliations:** 1 Department of Urology, Fudan University Shanghai Cancer Center, Shanghai, 200032 People’s Republic of China; 2 Department of Oncology, Shanghai Medical College, Fudan University, Shanghai, China; 3 Department of Urology, Tianjin Institute of Urology, The Second Hospital of Tianjin Medical University, 23 Pingjiang Road, Hexi District, Tianjin, 300211 People’s Republic of China; 4 Department of Urology, Peking University First Hospital, No. 8 Xishiku Dajie, Xicheng District, Beijing, 100034 People’s Republic of China; 5 Acornmed Biotechnology Co., Ltd, Beijing, 100176 People’s Republic of China; 6 Tianjin Key Laboratory of Urology, Tianjin Institute of Urology, The Second Hospital of Tianjin Medical University, Tianjin, China

**Keywords:** Liquid biopsy, Urine tumor DNA, Cancer biomarkers, Molecular diagnosis, Early detection, Urothelial carcinoma

## Abstract

Urine-based testing is promising for noninvasive diagnosis of urothelial carcinoma (UC) but has suboptimal sensitivity for early-stage tumors. Herein, we developed a multitarget urine tumor DNA test, UI-Seek, for UC detection and evaluated its clinical feasibility. The prediction model was developed in a retrospective cohort (*n* = 382), integrating assays for *FGFR3* and *TERT* mutations and aberrant *ONECUT2* and *VIM* methylation to generate a UC-score. The test performance was validated in a double-blinded, multicenter, prospective trial (*n* = 947; ChiCTR2300076543) and demonstrated a sensitivity of 91.37% and a specificity of 95.09%. The sensitivity reached 75.81% for low-grade Ta tumors and exceeded 93% in high-grade Ta and higher stages (T1 to T4). Simultaneous identification of both bladder and upper urinary tract tumors was enabled with sensitivities exceeding 90%. No significant confounding effects were observed regarding benign urological diseases or non-UC malignancies. The test showed improved sensitivities over urine cytology, the NMP22 test, and UroVysion FISH alongside comparable specificities. The single-target accuracy was greater than 98% as confirmed by Sanger sequencing. Post-surgery UC-score decreased in 97.7% of subjects. Overall, UI-Seek demonstrated robust performance and considerable potential for the early detection of UC.

**Supplementary Information:**

The online version contains supplementary material available at 10.1186/s12943-024-01974-4.

## Background

Urothelial carcinoma (UC) is one of the most common urological malignancies worldwide and mainly encompasses tumors of the bladder (BCa) and upper tract (UTUC) [1, 2]. Early detection of UC is strongly associated with a favorable prognosis, as the 5-year survival rate exceeds 90% for patients with localized tumors [3]. The most prevalent symptom of UC is hematuria, but less than 20% of patients are diagnosed with malignancy [4]. Cystoscopy combined with biopsy, the gold standard for diagnosis, is a costly and invasive procedure with low patient compliance [5]. Urine cytology is widely used as an adjunct to cystoscopy but has unsatisfactory sensitivity for detecting low-grade tumors and UTUC [1, 2]. The use of other approved urinary tests, including the NMP22 test and UroVysion FISH, is limited in routine practice predominantly due to their suboptimal accuracy and lack of demonstrable clinical benefits [6]. Thus, a noninvasive, convenient, and more effective tool for UC detection is urgently needed to complement the current screening strategy.

Currently, multiple urine-based tests based on genomic or epigenomic features have shown promising utility in UC diagnosis. UroSEEK presented advantages over cytology by incorporating profiles of mutations and copy number variants [7]. Bladder EpiCheck applied 15 methylation targets and demonstrated considerable accuracy in multiple studies [8]. AssureMDx combined methylation analysis with mutation analysis and showed the potential to triage hematuria patients [9]. Nonetheless, the limited amount of tumor cells shedding into the urine prevented the identification of UC at an early stage via urine-based tests [6, 10]. Recently, a novel multitarget utDNA (mt-utDNA) test involving assays of *FGFR3* and *TERT mutations* and methylation of *ONECUT2* and *VIM* was developed to facilitate the early detection of UC.

Herein, we reported the development of the mt-utDNA test and its clinical validation outcomes in a double-blinded, multicenter, prospective study. Overall, the test exhibited reliable and robust diagnostic ability in the clinical setting and exhibited its great potential as a supplement for the early detection and screening of UC in clinical practice.

## Results and discussion

### Study design

**Fig. 1:**
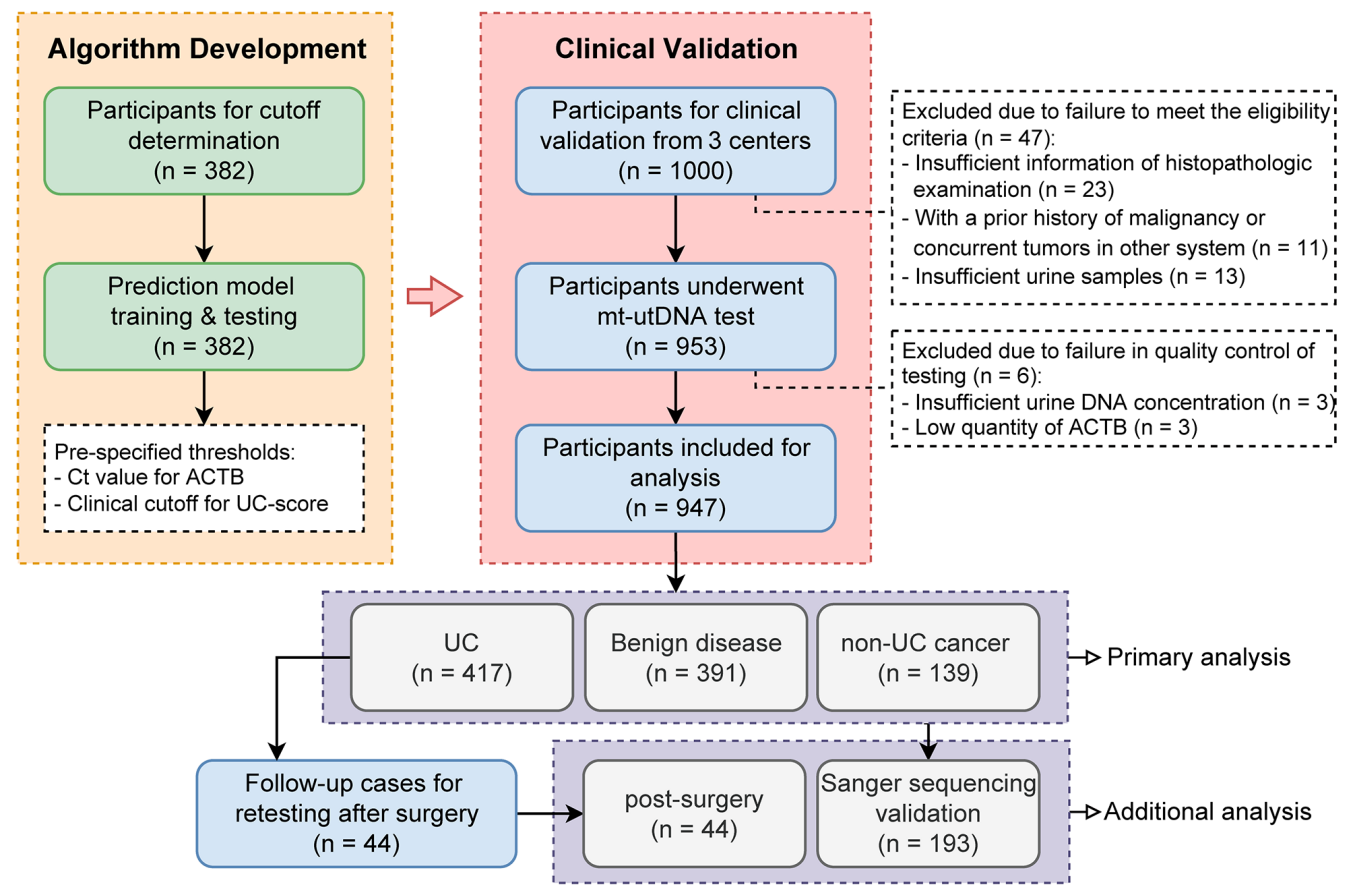
Flow diagram of algorithm development and clinical validation of the mt-utDNA test. A total of 1,329 participants were enrolled in this study. The algorithm development cohort included 382 individuals involved in the construction of the integrated prediction model and the determination of appropriate clinical cutoffs of the mt-utDNA test. The performance of the test was validated in the prospective validation cohort (*n* = 947) for UC detection according to the clinical diagnosis results. Sanger sequencing and postsurgical follow-up were performed to verify the results of the test. UC: urothelial carcinoma; mt-utDNA test: multitarget urine tumor DNA test

The current study consisted of two phases with independent cohorts: the algorithm development phase in a case-control cohort (*n* = 382) and the clinical validation phase in a double-blinded, multicenter, prospective trial (*n* = 947; Fig. 1). To our knowledge, this is the largest study of UC diagnosis that covers both BCa and UTUC. Participant enrollment and clinical and laboratory procedures are detailed in Supplementary Methods (Additional file 1).

### Algorithm development of the mt-utDNA test

The algorithm for the mt-utDNA test was established prior to the clinical trial (Additional file 3: Table S1). First, the cutoff values for the reference gene and single target were determined (Additional file 1: Supplementary Methods; Additional file 3: Table S2). Subsequently, to improve the accuracy and convenience in clinical applications, an integrated prediction model was constructed with the UC-score as the output. The model was trained (*n* = 252), and the cutoff was determined (i.e., UC-score ≥ 0 was considered positive) to balance the sensitivity and false-positive rate. Then, the established cutoffs were validated in the testing set (*n* = 130). The UC-score had an AUC of 0.9342, a sensitivity of 89.23%, and a specificity of 92.31% with the prespecified cutoff (Additional file 2: Fig. S2; Additional file 3: Table S3).

### Validation of the mt-utDNA test in a prospective clinical trial

In the clinical validation, 947 (94.7%) participants were ultimately included, consisting of 417 pathology-confirmed UC patients, 391 participants with benign urologic diseases, and 139 with non-UC cancers (Fig. 1; Additional file 3: Table S4).

**Fig. 2:**
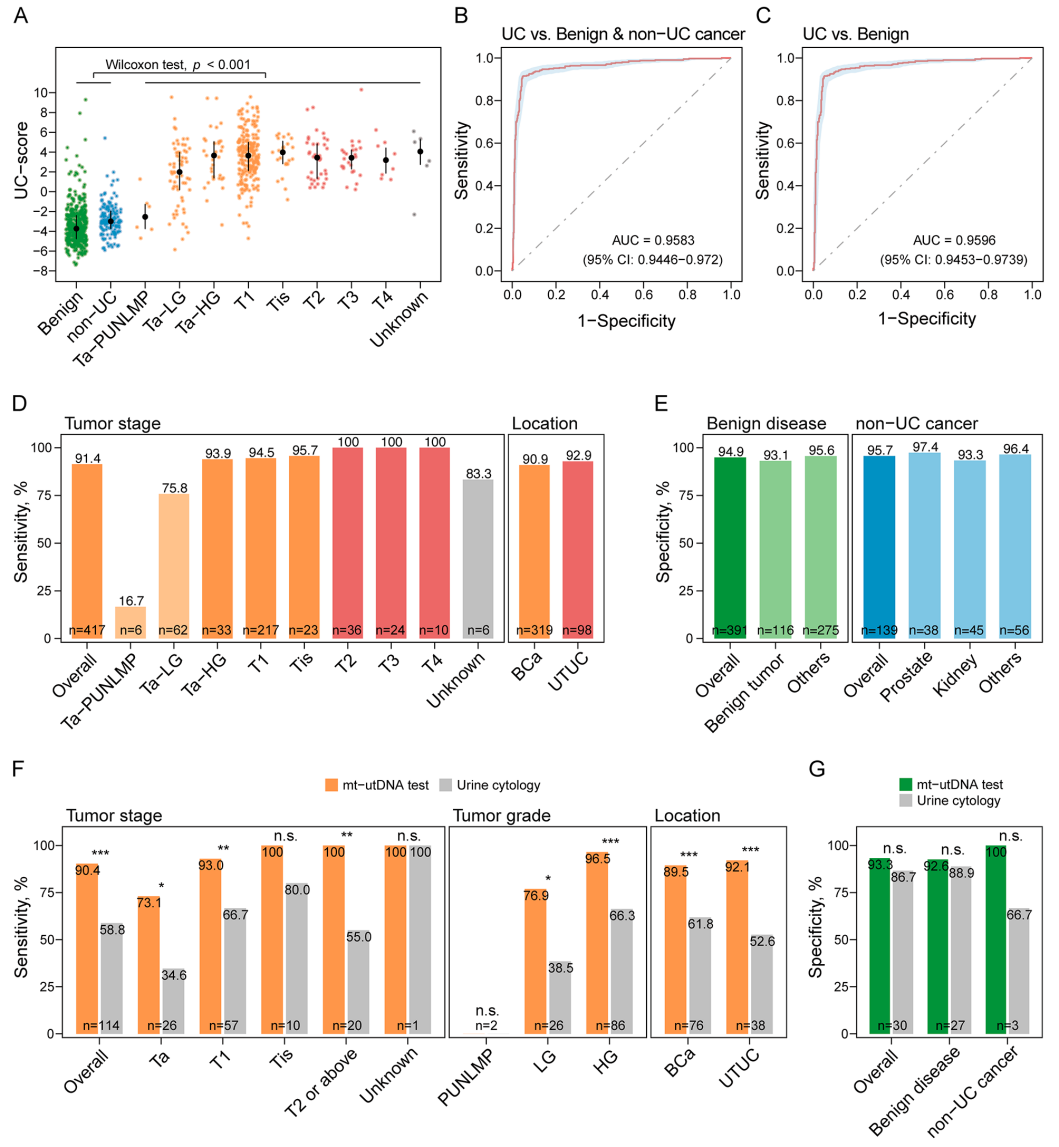
Performance of the mt-utDNA test in the clinical validation study. (**A**) Distribution of the UC-scores across pathological groups. The median values are depicted as black dots with the line range of the interquartile range. Statistical significance was assessed by the Wilcoxon rank sum test between the UCs and non-UCs. “benign” represented the urological benign disease group and “non-UC” represented the non-UC cancer group. (**B**) and (**C**) ROC curve plots of the mt-utDNA test for distinguishing UC patients from non-UC individuals, including those with benign urologic diseases or non-UC cancers (**B**), or from the subgroup of patients with benign urologic diseases (**C**). (**D**) Sensitivity of the mt-utDNA test for the indicated stage (left) or location (right) of UC tumors. Tumors of Ta stage were stratified as Ta-PUNLMP, Ta-LG and Ta-HG. (**E**) Specificity of the mt-utDNA test for the indicated types of benign urologic diseases (left) and non-UC cancers (right). (**F**) Sensitivity of the mt-utDNA test in UC patients with the indicated stage (left), grade (center), and location (right), in comparison with urine cytology. Statistical significance was assessed by Pearson’s Chi-squared test. (**G**) Specificity of the mt-utDNA test in non-UC participants with the indicated type of benign urologic diseases or non-UC cancers, in comparison with urine cytology. Statistical significance was assessed by Pearson’s Chi-squared test. Tumors combined with carcinoma in situ (CIS) were defined as Tis; UC, urothelial carcinoma; PUNLMP, papillary urothelial neoplasm of low malignant potential; LG, low grade; HG, high grade; AUC, area under the ROC curve; CI, confidence interval; BCa, bladder cancer; UTUC, upper tract urothelial carcinoma; *P < 0.05; **P < 0.01; ***P < 0.001; n.s., no significance

UC-scores were significantly greater in UC patients than in those with benign diseases or non-UC cancers (*p* < 0.001; Fig. 2A). UC-score increased with tumor grade and stage, but varied little among tumors located in the bladder, ureter, or renal pelvis (Fig. 2A; Additional file 2: Fig. S3). The area under the ROC curve (AUC) of the test was 0.9583 (Fig. 2B). The mt-utDNA test exhibited a sensitivity of 91.37% (381/417) in UC patients. The overall specificity was 95.09% (504/530), while it was 94.88% (371/391) for benign urologic diseases (AUC = 0.9596; Fig. 2C). The test demonstrated an outstanding consistency with the pathological diagnosis (κ = 0.8668). Additionally, the performance was stable among the three independent centers suggesting the robustness of the test (Additional file 3: Table S5 and S6).

Among UC patients, the detection rates increased with tumor stage and grade (*p* < 0.001; Additional file 3: Table S7), with sensitivities of 78.22%, 94.47%, and 100%, respectively, for Ta, T1, and T2 or above tumors. Notably, among Ta tumors, 75.81% of low-grade and 93.94% of high-grade tumors were identified (Fig. 2D), indicating that the test outperformed the single-marker assays reported in Oh’s and Deng’s studies [11, 12]. Given the high heterogeneity of UC tumors, these advantages in sensitivity, especially for early-stage tumors, were presumably attributed to the synergistic effect of multiple targets (Additional file 3: Table S8). Besides, the test enabled simultaneous detection of BCa and UTUC with a sensitivity exceeding 90% for both (Fig. 2D). The test showed widely low sensitivity to urological benign lesions and non-UC cancers (Fig. 2E; Additional file 3: Table S9), indicating strong resistance to common confounding factors. Overall, the mt-utDNA test was proven to be accurate and robust across a variety of patients suspected of UC in clinical settings.

The need for invasive, costly, and time-intensive examinations (cystoscopy, contrast-enhanced CT scans, etc.) to achieve a diagnostic conclusion is a significant issue associated with screening for UC. Impressively, the mt-utDNA test exhibited a positive predictive value (PPV) and negative predictive value (NPV) of 93.61% and 93.33%, respectively. Given the potential enrollment bias of this case-control trial, estimations of microscopic and gross hematuria populations (with an incidence of UC of 3.3% and 17%, respectively) were performed, and the NPVs were 99.69% and 98.17%, respectively (Additional file 2: Fig. S4A-B) [4]. Woldu et al. stratified hematuria patients into American Urological Association risk strata (low, intermediate, or high risk) based on sex, age, degree of hematuria, and smoking history [13], and reported the incidence of malignancy as 0.4%, 1.0%, and 6.3% in each group [14]. Correspondingly, the estimated post-test probability of UI-Seek positive subjects raised to 6.96%, 15.83%, and 55.60%, respectively (Additional file 2: Fig. S4C-E). The combination of the mt-utDNA test and other risk factors was expected to provide more accurate risk stratification. This could eventually increase clinical benefits by facilitating clinical decision-making and accelerating the referral of suspected UC patients.

Methodology comparisons of the mt-utDNA test with current urinary tests were conducted via head-to-head analysis. The sensitivity of the mt-utDNA test was remarkably superior to that of urine cytology (*p* < 0.001), the NMP22 test (*p* < 0.001), and UroVysion FISH (*p* = 0.039), while the specificity was comparable (Additional file 3: Table S10). A 2-fold improvement in sensitivity was observed for Ta stage (*p* = 0.012) and low-grade tumors (*p* = 0.011) compared with cytology (Fig. 2F). Indeed, the advantage was prevalent across subgroups of tumors of the indicated stages, grades, and locations (Fig. 2F). Comparisons of specificity showed similar tendencies, but no statistical significance was observed owing to the insufficiency of paired results (Fig. 2G). Overall, the mt-utDNA exhibited marked advantages in terms of sensitivity, particularly for low-grade and early-stage tumors.

The accuracy of each target was verified by Sanger sequencing, with a consistency ranging from 98.45 to 100% (Additional file 3: Table S11). Furthermore, in the post-surgery study conducted in randomly selected UC patients, 97.7% (43/44) of the subjects presented decreased UC-scores (*p* < 0.001; Additional file 3: Table S12; Additional file 2: Fig. S5), and 82.1% (32/39) experienced positive-to-negative conversion after resection, strongly suggesting that the positive results were specifically due to the signals released by the resected tumors. These results indicated that the UC-score might correlate with tumor burden and could imply the presence or residual of UC, which needed further confirmation in a long-term follow-up study.

Limitations existed in this study. First, the number of patients enrolled in the methodology comparisons was limited, hence sufficient power for full assessment in a representative population was lacking. Second, since the enrollment was based on a symptomatic population, the feasibility of the mt-utDNA test for screening in the asymptomatic population still requires in-depth evaluations in large-scale prospective studies. Other limitations included: (1) the clinical effectiveness of combining mt-utDNA test with additional risk factors (age, sex, hematuria, etc.) still needed to be validated in subsequent studies; (2) the long-term follow-up data was lacking; (3) the potential impact of random voided urine on the test needed further investigation; (4) since the study was performed in China, the generalizability for other populations remained to be assessed.

## Conclusions

In summary, this study revealed the effectiveness of the newly developed mt-utDNA test in the largest, multicenter, prospective clinical trial for UC detection. Its outstanding performance, especially the high sensitivity for detecting early-stage and low-grade tumors, highlights its potential as an aid for UC screening and diagnosis.

### Electronic supplementary material

Below is the link to the electronic supplementary material.

Supplementary Material 1

Supplementary Material 2

Supplementary Material 3

## Data Availability

The datasets analyzed in the current study are available upon reasonable request from the corresponding author.

## Abbreviations

AUC: Area under the ROC curve
BCa: Bladder cancer
CI: Confidence interval
CIS: Carcinoma in situ
HG: High grade
LG: Low grade
mt-utDNA test: Multitarget urine tumor DNA test
NPV: Negative predictive value
PPV: Positive predictive value
PUNLMP: Papillary urothelial neoplasm of low malignant potential
UC: Urothelial carcinoma
UTUC: Upper tract urothelial carcinoma
