# Supplementary material for: Clinical effectiveness of a multitarget urine DNA test for urothelial carcinoma detection: a double-blinded, multicenter, prospective trial
Source: Mol Cancer. 2024 Mar 19;23:57. doi: 10.1186/s12943-024-01974-4 (PMC10949661; doi:10.1186/s12943-024-01974-4)
Supplement: Supplementary file 1 — Supplementary Material 1 [file 12943_2024_1974_MOESM1_ESM.docx]

# Supplementary Methods

### ***Study Design***

This double-blinded, prospective clinical study of the multitarget urine tumor DNA (mt-utDNA) test was conducted parallelly in three clinical centers: Fudan University Shanghai Cancer Center (FUSCC), The Second Affiliated Hospital of Tianjin Medical University (2^nd^ HATMU), and Peking University First Hospital (PKUFH), following the Technical Guidelines for Clinical Trials of *in Vitro* Diagnostic Reagents (National Medical Products Administration, notice No. 72, released in 2021). The sample size of enrollment was estimated assuring the statistical power and representativeness of the consistency assessment (see section “Sample Size Estimation”). Written informed consent was obtained from all participants. Urine samples were collected by the physicians unaware of the pathological results. The performers of the mt-utDNA test were kept blinded to the diagnosis information while the physicians or the subjects were not informed of the test results. Participants were grouped according to the clinical diagnosis as the UC group and non-UC control group, the latter compromising benign urologic diseases and non-UC cancers. Sensitivity and specificity were calculated to evaluate the agreement of the mt-utDNA test compared with the clinical reference results (via histopathologic examination and/or comprehensive diagnosis). This clinical trial was registered on the website of the Chinese Clinical Trial Registry (www.chictr.org.cn; registration number ChiCTR2300076543). The Standards for Reporting of Diagnostic Accuracy (STARD) criteria were followed in this study where applicable [[1](#_ENREF_1), [2](#_ENREF_2)].

### ***Sample Size Estimation***

For the assessment of the diagnostic consistency between the mt-utDNA test and the clinical reference standards, the sample size was estimated based on the following equation:

$$n=\frac{{[Z_{1-\alpha/2}\sqrt{P_{0}\left( 1-P_{0} \right)}+Z_{1-\beta}\sqrt{P_{T}\left( 1-P_{T} \right)}]}^{2}}{{(P_{T}-P_{0})}^{2}}$$

where $n$ is the estimated sample size, $Z_{1-\alpha/2}$, $Z_{1-\beta}$ is the standard normal deviate according to the desired confidence level of type I error (α) and power (1-β), $P_{T}$ denotes the pre-determined value of sensitivity (or specificity) of the test to be evaluated, and $P_{0}$ denotes the clinically acceptable criterion. The hypothetical 95% lower boundary of sensitivity and specificity of the clinical criterion was 75% and 85%, respectively. According to the preliminary exploratory study (unpublished data), the estimate was 85% for sensitivity and 90% for specificity of the mt-utDNA test. With the two-sided type I error set at 0.05 and the power at 0.8, the minimum sample size for the positive group (UC cases) and the negative group (non-UC controls) was 132 and 363, respectively. Considering the dropout and loss rate during the clinical study together with the request that positive cases should encompass all tumor stages and subtypes, the requirement of enrollment was 400 for both UC cases and non-UC controls.

### ***Study Population***

Prior to the clinical validation, a retrospective cohort of 382 individuals was enrolled independently for the establishment of the predictive algorithm. In the prospective clinical validation study, a total of 1000 participants were consecutively recruited at 3 hospitals from February 2023 to August 2023, including individuals suspected of UC (with symptoms like hematuria, abnormal imaging, etc.) as well as subjects affected with non-UC cancers (prostate, kidney, etc.). All participants were aged ≥18 years. The participants were excluded in the following conditions: i) history of malignancy, ii) suspected of multiple primary tumors, iii) unqualified or failure of the test, iv) insufficient clinical information (demographic data, pathological diagnosis results, etc.). The mutation and methylation status of each single target were verified via Sanger sequencing among 193 subjects. Separately, 44 UC patients who had undergone curative-intent surgery via transurethral resection (TUR) or radical cystectomy were randomly selected from three clinical centers and subjected to a post-surgery follow-up study. They were sampled and tested a second time within 2 months after resection. The median intervals after surgery were 15 days (range, 1-49 day; IQR, 9-24 days; Additional file 3: Table S1).

### ***Clinical Procedures***

After providing written informed consent, all eligible participants were required to provide a voided morning urine sample prior to surgical resection or treatment. Each of the samples was assigned a unique identifier for blinded testing. As the reference standard for diagnosis, the result of histopathological examination on surgical tissue specimens was utilized to categorize each participant. UC patients were staged and graded according to the 8th edition American Joint Committee on Cancer (AJCC) Cancer Staging Manual and the 2004/2016 WHO grading system [[3](#_ENREF_3), [4](#_ENREF_4)]. For non-UCs who did not undertake surgery, the identification was based on the results of comprehensive diagnosis, including but not limited to urine cytology, cystoscopy/ureteroscopy, ultrasound examination, cross-sectional imaging, laboratory tests, etc. The corresponding clinical characteristics of patients’ age and sex were also recorded. The available results of urine cytology, the NMP22 test, and UroVysion FISH were retrieved for the comparative analysis.

### ***Sample Processing***

Approximately 100-200 mL voided morning urine samples of the participants were collected and stabilized using a dispensable urine storage tube (Acornmed Medical Devices, Tianjin, China) containing DNA preservation buffer and stored at 4 ℃. The urinary cell pellets were separated within 24 hours after voiding by centrifugation at 1,600 rpm for 20 min with the supernatant removed, and then washed twice and resuspended with PBS. The urine DNA from the pellets was isolated using the DNA extraction and purification kit (Acornmed Medical Devices, Tianjin, China) according to the manufacturer’s instructions. Briefly, 200 μL of the resuspended sample was digested with Proteinase K and lysis buffer and incubated at 70℃ for 10 min, then transferred to the purification column after adding 200 μL of absolute ethanol. Purified DNA was eluted in 53 μL elution buffer. No template control (NTC) was added in each run to assess the potential contamination during the test. DNA concentration was measured by Qubit 4.0 (Thermo Fisher Scientific, Shanghai, China), and participants with DNA concentration < 2.5 ng/mL were excluded. Extracted DNA was stored at -20 ℃ until further processing.

Extracted DNA was split, and one portion was subjected to sodium bisulfite to convert unmethylated cytosine to uracil while leaving methylated cytosine unchanged, using the methylation pre-processing kit (Acornmed Medical Devices, Tianjin, China). Briefly, sample DNA was added into the bisulfite treatment reaction and incubated at 98 ℃ for 8 min followed by 54 ℃ for 1 hr. Afterwards, the bisulfite-treated DNA (bis-DNA) was transferred to the purification column for binding and washing, and eventually eluted out in 12 μL elution buffer. NTC and positive control were treated parallelly with samples in each run. Bis-DNA was stored at 2~8 ℃ prior to test or frozen at -20 ℃ within 3 days.

### Quantitative Real-time PCR (qPCR) and Quantitative Methylation-specific PCR (qMSP) ***Testing and Analyzing***

The multitarget utDNA test, UI-Seek, manufactured by Acornmed Medical Devices Co., Ltd. (Tianjin, China), consisted of molecular assays for mutant *TERT* and *FGFR3*, aberrantly methylated *ONECUT2* and *VIM* promoter regions, and β-actin (*ACTB*, as internal reference). Two sites of *TERT* promoter region (*TERT*-M1 and *TERT*-M2) and two sites of *FGFR3* (*FGFR3*-M1 and *FGFR3*-M2) were amplified in the unconverted DNA following a procedure of qPCR. Meanwhile, qMSP was employed to quantitatively detect *ONECUT2* and *VIM* methylation status in bis-DNA samples. *ACTB* was set as the internal control for both qPCR and qMSP. PCR reactions were performed on the Applied Biosystems 7500 Real-Time PCR System (Thermo Fisher Scientific, Shanghai, China) under the following thermocycling program: 37 ℃ for 3 min; 98 ℃ for 2 min; 45 cycles of denaturing at 98 ℃ for 20 s and annealing/extension at 62 ℃ for 45 s. The fluorescent signals were collected at the annealing/extension step. The quantification cycle (Cq) values, which represented the relative quantity of the detected targets and the internal reference gene (ACTB), were measured by FAM, CY5, and VIC signals separately after setting the baseline and threshold of amplification curves. All valid samples should satisfy the quality control requirement of qPCR Cq value of ACTB ≤ 28 and qMSP Cq value of ACTB ≤ 31. Representative amplification curves of selected regions of targets and internal control in a randomly selected urine sample, the standard (STD) positive control, and NTC are shown in Fig. S1 (Additional file 2).

For all valid samples, undetermined Cq values were arbitrarily assigned as 45 and ΔCq was calculated between the Cq value of each target and the corresponding Cq value of *ACTB* (Cq_Target_-Cq_ACTB_). The value of ΔCq was used as the quantitative measurement to dichotomize the test result of each single target, and subsequently incorporated into a validated, pre-specified logistic-regression algorithm referred to as UC-score Calculator (Acornmed Medical Devices, Tianjin, China). The calculated UC-scores, as the output indicating the risk of the presence of UC tumors, were used to dichotomize the tested subjects as “positive” (UC-score ≥ 0) or “negative” (UC-score < 0).

### ***Determination of the Appropriate Clinical Cutoffs.***

Prior to the onset of the clinical trial, the appropriate cutoff value for each single target and the integrated prediction model was determined in a retrospective case-control cohort consisting of 382 participants (196 UCs, 171 benign diseases, and 15 non-UC cancers; Additional file 3: Table S1). Firstly, the one-side percentile method was applied to define the threshold of ACTB Cq value as the quality control criteria of sufficient input DNA or bis-DNA. The Cq threshold of 28 was set for valid tests of qPCR based on the data that 95% of subjects in a group of 348 measurements presented ACTB Cq values ≤ 28 in the qPCR reaction. Similarly, the Cq threshold of 31 was set for ACTB in qMSP tests based on 696 measurements (Additional file 3: Table S2). Next, receiver operating characteristic (ROC) curve analysis was implemented to assess the ΔCq value for each target to serve as the positive cutoff. The optimal cutoff value was determined by the maximized Youden index (sensitivity + specificity – 1) as ΔCq ≤ 8 for *ONECUT2* and *VIM*, ΔCq ≤ 12 for *FGFR3*-M1 and *TERT*-M1, and ΔCq ≤ 11 for *FGFR3*-M2 and *TERT*-M2.

Thereafter, to achieve an expected performance and improve the convenience of clinical application, the measurements of each single target and the internal reference were integrated as the UC-score via a logistic-regression algorithm:

$$UC\text{-}score= \sum_{k=1}^{n} a_{k}\times\left( -{\Delta Cq}_{k} \right)+b$$

where $k$ represents the indicated target, $a_{k}$ represents the coefficients obtained by training, ${\Delta Cq}_{k}$ represents the ΔCq value of the k target, and $b$ represents the constant obtained by training. The prediction model was trained in 252 participants (“Training set”; Additional file 3: Table S1) and the optimal cutoff value of UC-score was determined as with balanced sensitivity and false positive rate (i.e., UC-score ≥ 0 as “positive”, UC-score < 0 as “negative”). Then, the established clinical cutoffs were validated in an independent testing set (Additional file 3: Table S1). The performance of the prediction model in the development phase is summarized in Fig. S2 (Additional file 2) and Table S3 (Additional file 3).

### ***Statistical Analysis***

The sensitivity and specificity with 95% confidence interval (CI) were used to assess the diagnostic accuracy of the mt-utDNA test. Cohen’s kappa statistic, κ, was used to measure the consistency with reference diagnosis results. κ ≥ 0.75 suggests that the test has a substantial diagnostic consistency. Receiver operating characteristic (ROC) curve analysis was implemented using the R package pROC and the area under the ROC curve (AUC) with 95% CI was calculated. Wilcoxon rank sum test or Kruskal-Wallis test were performed for inter-group comparisons of UC-score. Paired Wilcoxon rank sum test was used in matched samples in the follow-up study. Pearson’s Chi-squared test or Fisher’s exact test was applied to evaluate the correlation of test results with demographic and clinical characteristics. McNemar's test was conducted in the head-to-head methodology comparisons. All hypothesis tests were two-sided with a *P* < 0.05 considered statistically significant. All statistical analysis and data visualizations were performed using R software version 4.2.2 (The R Foundation; <https://www.r-project.org/>).

# References

1. Jérémie FC, Daniël AK, Douglas GA, David EB, Constantine AG, Lotty H, et al: STARD 2015 guidelines for reporting diagnostic accuracy studies: explanation and elaboration. BMJ Open. 2016;6:e012799.

2. Bossuyt PM, Reitsma JB, Bruns DE, Gatsonis CA, Glasziou PP, Irwig L, et al: STARD 2015: an updated list of essential items for reporting diagnostic accuracy studies. BMJ. 2015;351:h5527.

3. AJCC cancer staging manual*.* 8th ed. Chicago IL: American Joint Committee on Cancer, Springer; 2017.

4. WHO classification of tumours of the urinary system and male genital organs*.* 4th ed. Lyon: International agency for research on cancer; 2016.
