# Supplementary material for: Clinical effectiveness of a multitarget urine DNA test for urothelial carcinoma detection: a double-blinded, multicenter, prospective trial
Source: Mol Cancer. 2024 Mar 19;23:57. doi: 10.1186/s12943-024-01974-4 (PMC10949661; doi:10.1186/s12943-024-01974-4)
Supplement: Supplementary file 2 — Supplementary Material 2 [file 12943_2024_1974_MOESM2_ESM.docx]

# Supplementary Figures


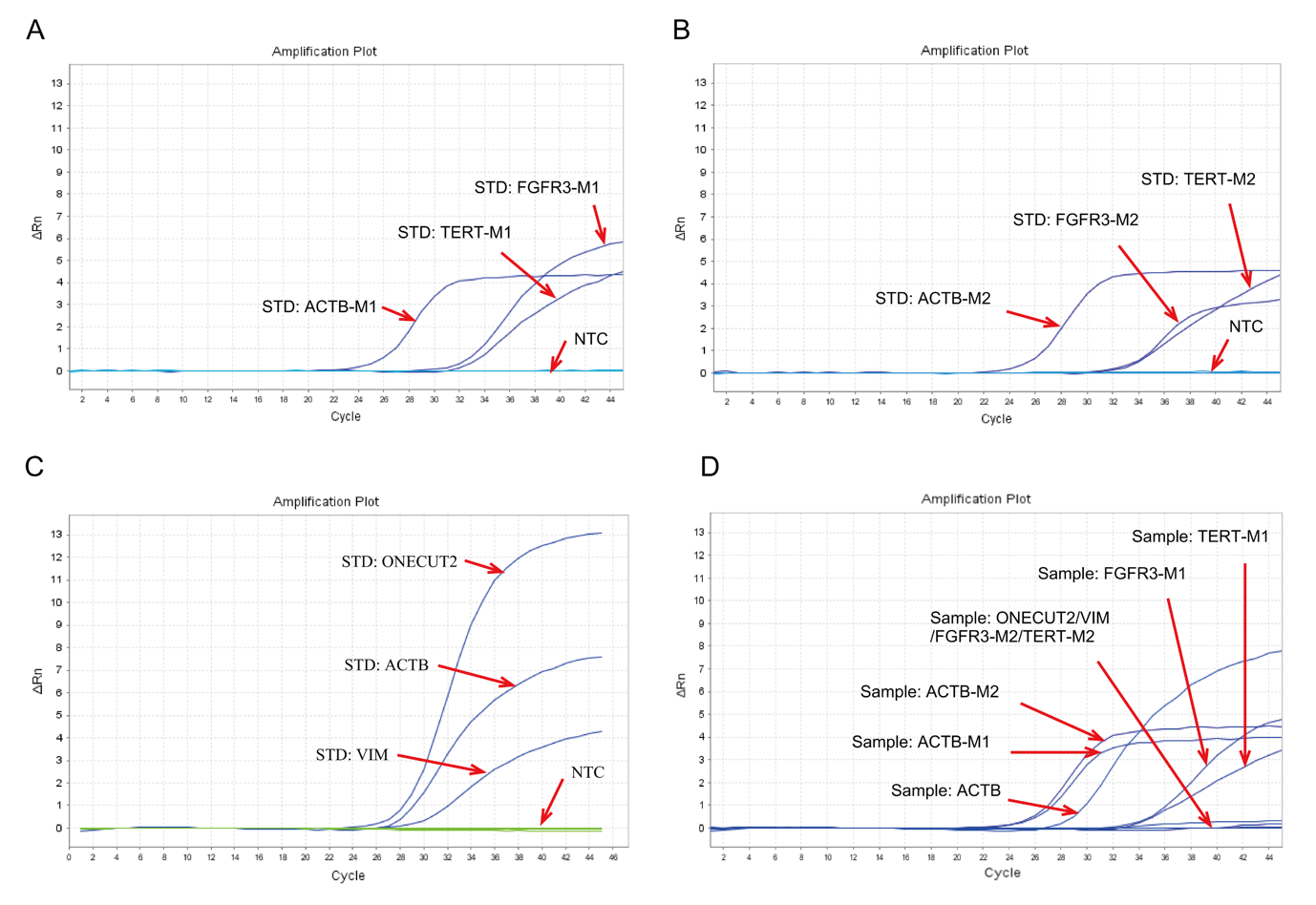


Fig. S1. Amplification curves by real-time qPCR and qMSP.

**(A)** Amplification curves of *ACTB*, mutated *TERT*-M1 and *FGFR3*-M1 in positive standard reference DNA (STD) and in negative control (NTC).

**(B)** Amplification curves of *ACTB*, mutated *TERT*-M2 and *FGFR3*-M2 in STD and in NTC.

**(C)** Amplification curves of *ACTB*, methylated *ONECUT*2 and *VIM* in STD and in NTC.

**(D)** Amplification curves of *ACTB*, mutated *TERT*-M1 and *FGFR3*-M1, unmutated *TERT*-M2 and *FGFR3*-M2, and unmethylated *ONECUT2* and *VIM* in a random sample of urine DNA.

The x axis indicates PCR cycles from 1 to 45. Rn, fluorescence intensity of normalized reporter. STD, positive standard reference; NTC, no template control


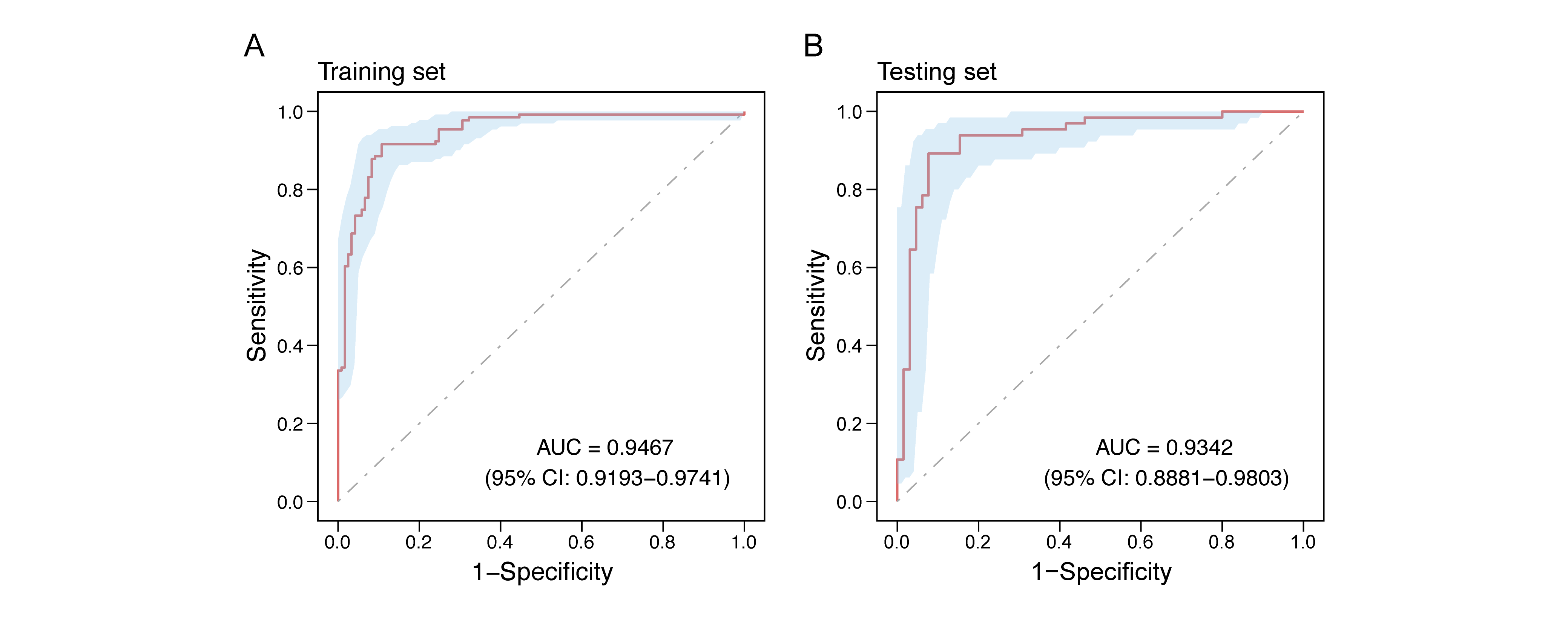


Fig. S2. ROC curve plots for algorithm development cohort.

**(A) and (B)** ROC plot of mt-utDNA test in training set (**A**, n = 252) and in testing set (**B**, n = 130).

AUC, area under the ROC curve; CI, confidence interval


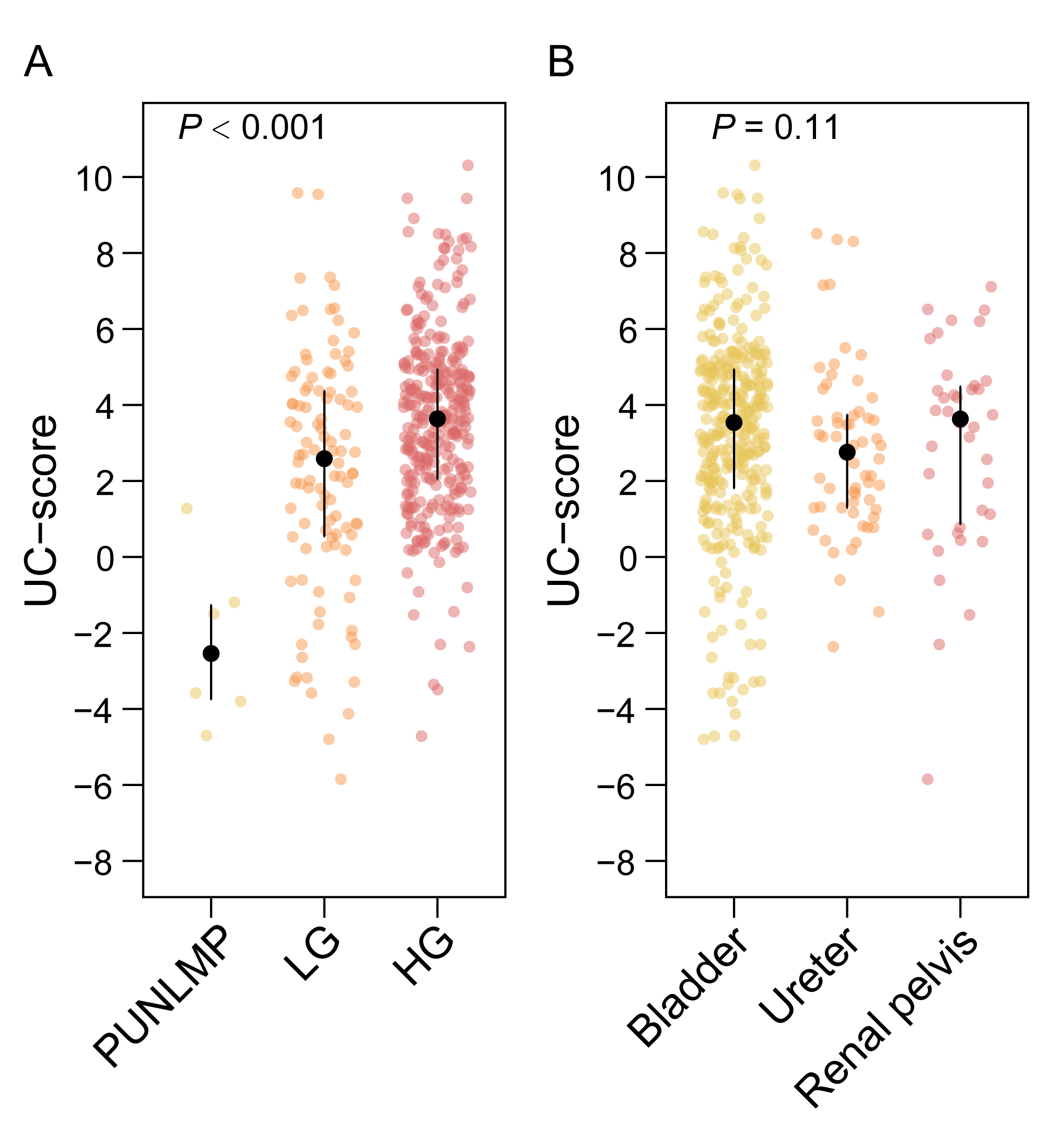


Fig. S3**.** Distribution of the UC-scores across different pathological groups.

**(A) and (B)** Distribution of the UC-scores with indicated grade **(A)** and location **(B)** of tumors.

The median values were depicted as dots in black with line range of interquartile range (IQR). Statistical significance was assessed by Kruskal-Wallis test. PUNLMP, papillary urothelial neoplasm of low malignant potential; LG, low grade; HG, high grade


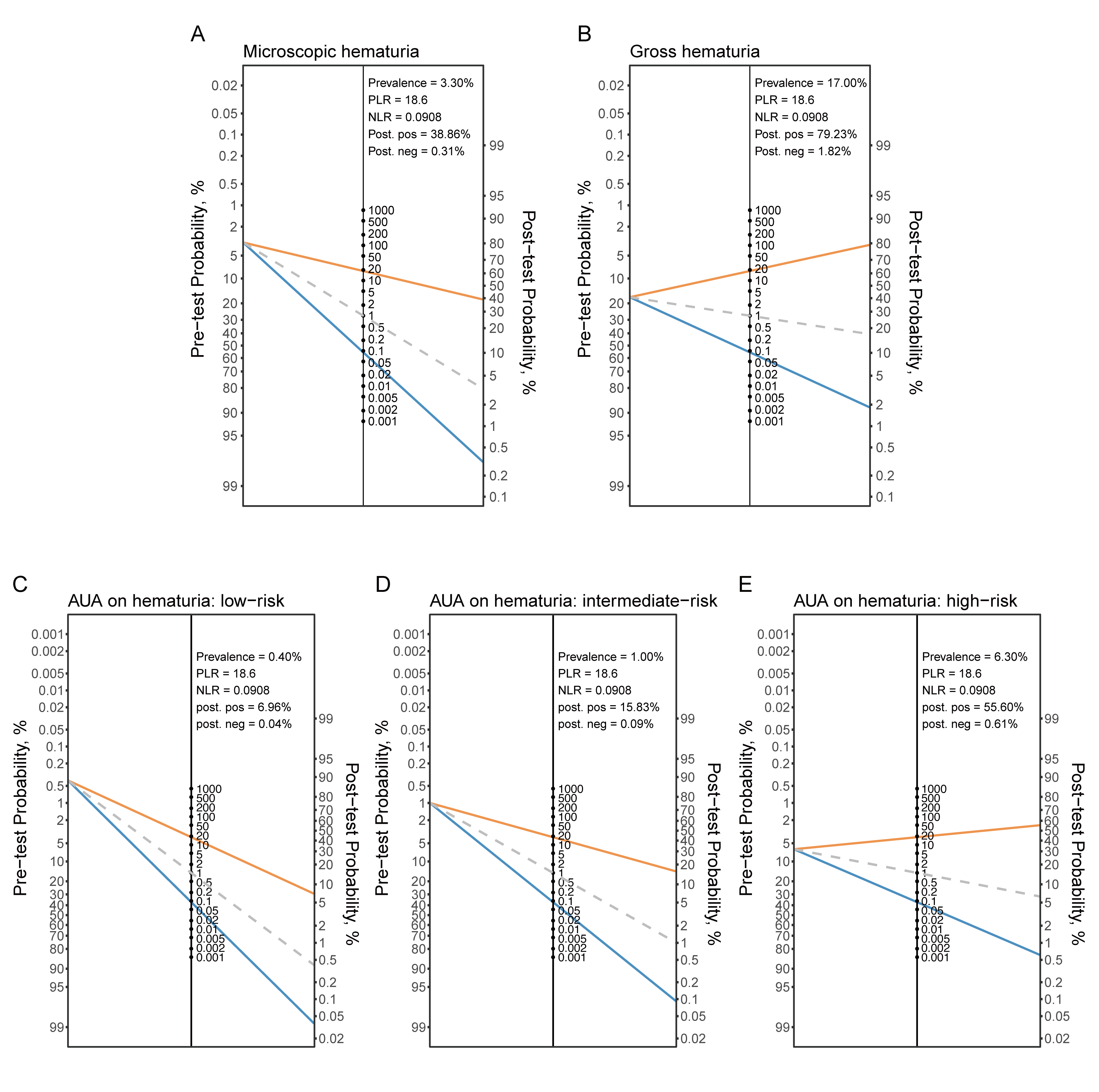


Fig. S4. Fagan nomogram of hematuria population risk stratification. Estimations of the post-test probability of UC for the population of microscopic hematuria (A), gross hematuria (B), low- (C), intermediate- (D), high-risk group (E) according to AUA guideline on hematuria.

Prevalence denoted the incidence of malignancy in indicated population. Estimations were based on a sensitivity of 91.37% and a specificity of 95.09% of the mt-utDNA test in this study. PLR, positive likelihood ratio; NLR, negative likelihood ratio.


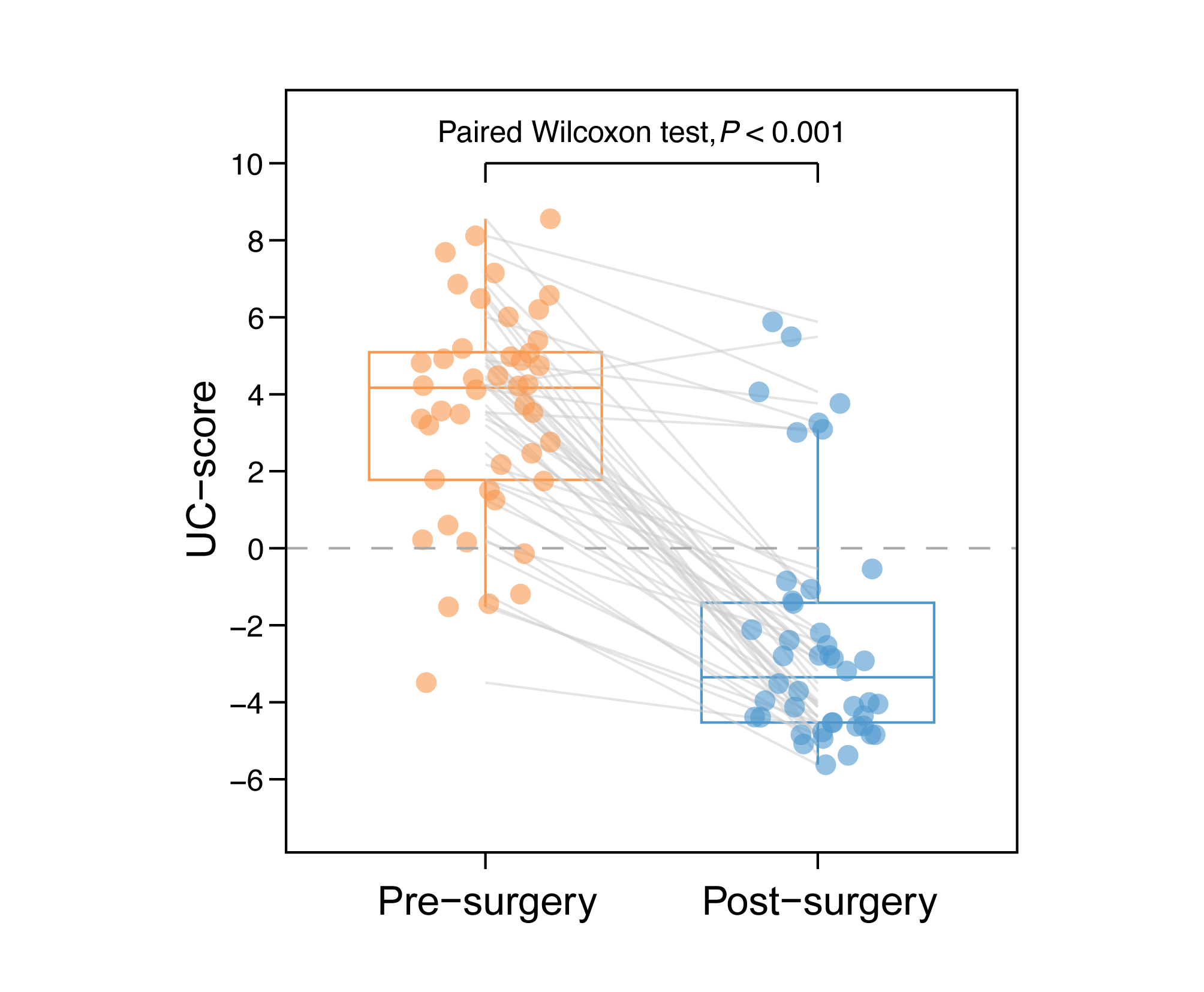


Fig. S5. Dynamic of UC-score after surgical resection. Statistical significance was assessed by paired Wilcoxon rank sum test between the matched pre-surgery and post-surgery scores.
